# Diagnostik von Lungenerkrankungen anhand kleiner Präparate: Zytologien und Biopsien

**DOI:** 10.1007/s00292-025-01521-y

**Published:** 2025-12-08

**Authors:** Tereza Losmanova, Marie Maillard, Ekkehard Hewer, Sabina Berezowska

**Affiliations:** 1 Institut für Gewebemedizin und Pathologie, Universität Bern, Murtenstrasse 31, 3008 Bern, Schweiz; 2 Department of Laboratory Medicine and Pathology, Institute of Pathology, Lausanne University Hospital and University of Lausanne, Lausanne, Schweiz

**Keywords:** Lungenpathologie, Zytologie, Prädiktive Marker, Digitale Diagnostik, WHO-Klassifikation, Pulmonary pathology, Cytology, Predictive biomarkers, Digital diagnostics, WHO classification

## Abstract

Dieser Beitrag gibt einen praxisnahen Überblick darüber, wie thorakale Erkrankungen zuverlässig anhand kleiner Präparate beurteilt werden können. Er zeigt, wie sich zytologische und histologische Verfahren sinnvoll ergänzen, warum eine sorgfältige Präanalytik und Materialsteuerung entscheidend sind und wie Zusatzuntersuchungen mit Augenmaß eingesetzt werden, um Gewebe zu schonen. Der Artikel greift aktuelle Entwicklungen wie digitale Auswerteverfahren auf und formuliert alltagstaugliche Empfehlungen – inklusive typischer Fallstricke und Hinweise zur Ergebnisinterpretation.

## Abstract (en)

This article provides a practice-oriented overview of how thoracic diseases can be reliably assessed using small specimens. It demonstrates how cytologic and histologic methods complement each other, why careful preanalytical handling and specimen triage/tissue stewardship are critical, and how ancillary studies can be used judiciously to conserve tissue. The article also addresses current developments such as digital analysis workflows and offers practical recommendations—including common pitfalls and guidance on interpreting results.

## Lernziele

Nach der Lektüre dieses Beitrags …

- kennen Sie die Methodik der Untersuchung kleiner Lungenbiopsien und zytologischer Präparate sowie deren Einsatzmöglichkeiten und Grenzen;
- sind Sie mit der Nomenklatur neoplastischer Erkrankungen an kleinen Präparaten vertraut;
- verstehen Sie die gezielte Anwendung immunhistochemischer Marker zur Diagnostik von Lungentumoren an kleinen Präparaten;
- kennen Sie die wichtigsten Überlegungen zur Abklärung nichtneoplastischer Lungenerkrankungen an Biopsien und Zytologien.

## Fallbeispiel

Bei einer 62-jährigen Patientin wurden in der Positronenemissions‑/Computertomographie (PET/CT) ein **pulmonaler Rundherd**

pulmonaler Rundherd

sowie vergrößerte Lymphknoten der ipsilateralen Stationen 11 und 7 nachgewiesen. Daraufhin erfolgten eine Bronchusbiopsie sowie eine Staging-Untersuchung mittels **endobronchialer Sonographie**

endobronchialer Sonographie

(EBUS). In den Lymphknoten zeigte sich zytomorphologisch ein nichtkleinzelliges Karzinom.

**Abb. 1:**
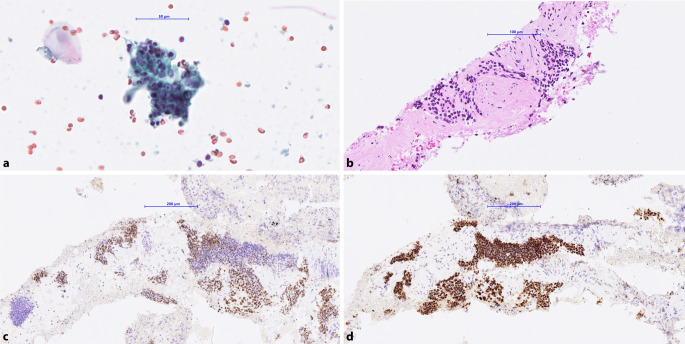
Bronchoskopische Feinnadelaspiration (FNA). **a** Zytomorphologie. **b** Histomorphologie im Zellblock. **c** TTF-1-Immunhistochemie. **d** p40-Immunhistochemie

Zur weiteren Typisierung wurde eine **immunhistochemische Untersuchung**

immunhistochemische Untersuchung

des TTF-1- und p40-Status durchgeführt. Die Tumorzellen zeigten eine positive Reaktion gegen p40 sowie mehrheitlich auch für TTF‑1 in denselben Zellpopulationen (Abb. 1). Aufgrund dieses selten auftretenden Expressionsmusters wurde die Diagnose eines nichtkleinzelligen Lungenkarzinoms (NSCLC) mit bilineärer („bi-lineage“) Differenzierung gestellt [1]. Ergänzend wurde eine **molekularpathologische Analyse**

molekularpathologische Analyse

mittels Next Generation Sequencing (NGS) indiziert. Für diese standen im EBUS-Material ausreichend Tumorzellen zur Verfügung.

## Methodische Aspekte

### Optimierte Verwendung von spärlichem Material

In Zeiten prädiktiver Marker ist eine optimale Nutzung und Auswahl des bestgeeigneten Materials entscheidend – insbesondere durch die kombinierte Anwendung von Zytologie und Histologie. Die Mehrheit der Patient:innen mit Lungenkarzinom wird nach wie vor in fortgeschrittenen Stadien diagnostiziert, wobei oft nur kleine zytologische oder histologische Präparate zur Verfügung stehen. Auch bei nichtneoplastischen Lungenerkrankungen ergänzen sich beide Methoden sinnvoll – insbesondere bei der Differenzierung der Zellzahl in der **bronchoalveolären Lavage**

bronchoalveolären Lavage

(BAL), die eine wichtige Rolle in der Diagnostik interstitieller Lungenerkrankungen spielt.

Das Motto der Zytologie lautet: *„doing more with less.“* Obwohl häufig angenommen wird, dass zytologisches Material weniger Gewebe enthält, trifft das nicht zwingend zu. Zytologie und Histologie sind komplementäre Methoden und werden während der Bronchoskopie oft parallel gewonnen.

Die Technik der **Feinnadelaspiration**

**Abb. 2:**
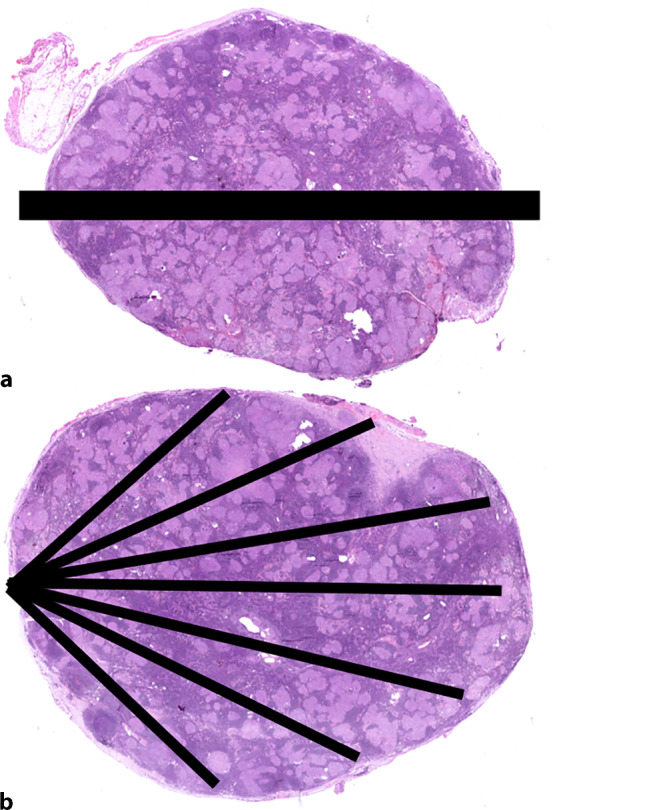
Sampling von Lymphknotengewebe mittels Biopsie (**a**) und Feinnadelaspiration (FNA, **b**)

Feinnadelaspiration

(FNA) ermöglicht durch fächerförmige Nadelbewegungen eine Probenentnahme aus einer größeren Fläche und eignet sich besonders gut für die Beurteilung mediastinaler Lymphknoten (Abb. 2).

Zudem erlaubt die Zytologie eine schnellere Bearbeitung – ein entscheidender Vorteil in akuten Situationen etwa zur raschen Differenzierung zwischen kleinzelligem Lungenkarzinom (SCLC), NSCLC und anderen Entitäten.

Färbungen zytologischer Ausstriche lassen sich in 2 Hauptgruppen unterteilen:

- Färbungen alkoholfixierter Ausstriche, gefärbt in der Regel mittels Papanicolaou(PAP)-Methode;
- Färbungen luftgetrockneter Ausstriche, gefärbt z. B. mittels Romanowsky, May-Grünwald-Giemsa (MGG) bzw. geeigneter kommerzieller Schnellfärbereagenzien.

Die Wahl der **Färbemethode**

Färbemethode

ist meist landes- bzw. institutsabhängig. Jede Färbung bietet spezifische Vorteile. Die PAP-Methode ermöglicht eine besonders gute Beurteilung von Kerndetails und Keratinisierung, Romanowsky-Färbungen heben zytoplasmatische Details sowie den Hintergrund besser hervor. In manchen Instituten werden beide Färbemethoden komplementär eingesetzt, was eine umfassendere diagnostische Beurteilung erlaubt. Letztlich richtet sich die Entscheidung jedoch oft nach lokalen Standards und den Erfahrungen des jeweiligen Teams.

Die zusätzliche Anfertigung eines **Zellblocks**

Zellblocks

hängt von der Art der Materialgewinnung ab. Wenn eine Nadelspülung – z. B. in NaCl – vorliegt, kann daraus direkt oder nach vorheriger zytologischer Beurteilung ein Zellblock hergestellt werden (insbesondere bei FNA- bzw. EBUS-Methoden). Zellblöcke können hinsichtlich Aufarbeitung und diagnostischer Verwertbarkeit den histologischen Paraffinblöcken weitgehend gleichgestellt werden und eignen sich gleichermaßen für immunhistochemische sowie molekularpathologische Analysen. Liegt das Material ausschließlich in Form von **Ausstrichen**

Ausstrichen

vor, können größere Gewebsfragmente vom Objektträger abgekratzt und zur Zellblockherstellung verwendet werden. Die Zytomorphologie ist dabei teilweise beeinträchtigt und der Erfolg ist nicht in allen Fällen gegeben.

In der **Nomenklatur**

Nomenklatur

wird zwischen Immunhistochemie (IHC) und Immunzytochemie (ICC) unterschieden. Die IHC wird am formalinfixierten, paraffineingebetteten Material (FFPE) – zumeist am Zellblock – analog zu histologischen Präparaten durchgeführt. Dabei ist sowohl die Art der allfälligen **Vorfixierung**

Vorfixierung

(z. B. bei Ergüssen mit Alkohol, wie es in einigen Instituten üblich ist) als auch die Methode der Zellblockanfertigung zu beachten; empfehlenswert ist eine vorgängige Testung mit unterschiedlichen **Antikörpern**

Antikörpern

[2]. Die ICC hingegen bezieht sich auf Färbungen, die an zuvor entfärbten zytologischen Ausstrichen oder ungefärbter **flüssigkeitsbasierter Zytologie**

flüssigkeitsbasierter Zytologie

(„liquid-based cytology“, LBC) durchgeführt werden. Aufgrund der variablen Vorfixierung dieser Materialien ist eine separate Revalidierung der verwendeten Antikörper erforderlich. Obwohl in der Praxis eher selten angewendet können auch immunzytologische Färbungen grundsätzlich für die Testung prädiktiver Marker geeignet sein [3, 4].

Eine zentrale Entscheidung betrifft die gezielte **Materialtriage**

Materialtriage

für die molekularpathologische Diagnostik. Sowohl zytologische als auch histologische Proben sind hierfür geeignet – entscheidend ist in beiden Fällen der **Tumorzellgehalt**

Tumorzellgehalt.

Besondere Aufmerksamkeit verdient dabei das EBUS-Material: Hier kann eine starke Beimischung lymphatischer Zellen zu einem reduzierten Tumorzellgehalt führen.

Ein interessantes Material für molekulare Untersuchungen ist der – im Wesentlichen zellfreie – **Überstand**

Überstand,

der sich aus der Zentrifugation zytologischer Proben ergibt. Verschiedene Studien haben gezeigt, dass sich in diesem Material, das traditionell verworfen wurde, regelmäßig ausreichend **Tumor-DNA**

Tumor-DNA

und -RNA finden, um die erforderlichen **Panelsequenzierungen**

Panelsequenzierungen

durchzuführen [5]. So kann z. B. der zellfreie Überstand von Feinnadelpunktaten oder Ergüssen zur Sequenzierungen verschiedener Panels verwendet und dadurch der Verbrauch von Schnitten des Zellblocks minimiert werden.

#### Merke

Molekulare Analysen sind auch an zytologischen Präparaten und sogar am zellfreien Überstand möglich.

### Korrelation mit klinischer Symptomatik, Bildgebung und Bronchoskopie

Wie auch in anderen komplexen diagnostischen Situationen ist eine effiziente und offene Kommunikation mit allen beteiligten Disziplinen für eine bestmögliche Diagnostik von Lungenerkrankungen gerade an kleinen Präparaten von essenzieller Bedeutung. In der Erfahrung der Autoren tragen insbesondere Bronchoskopie und EBUS wesentlich dazu bei, dass erfahrene Untersucher ihre differenzialdiagnostische Einschätzung präzisieren und der Pathologie relevante Zusatzinformationen bereitstellen können.

### Techniken zur Gewebegewinnung kleiner Biopsien/Zytologien

#### Sputum

- Nicht-invasive Untersuchungsmethode.
- Wird heute nur noch selten eingesetzt, kann jedoch eine Alternative für Patient:innen darstellen, bei denen eine Bronchoskopie nicht möglich ist.
- Die diagnostische Sensitivität ist insgesamt eher gering.

#### Exfoliative Methoden, Bürstenzytologie und Bronchialspülung

- Höhere Sensitivität im Vergleich zur Sputumuntersuchung.
- Werden meist ergänzend zu anderen diagnostischen Methoden eingesetzt.
- Für die Anwendung der Bürstenzytologie ist eine endoskopisch sichtbare Läsion erforderlich.

#### Bronchoalveoläre Lavage (BAL)

- Segmentale Spülung mit sterilem NaCl bei fest positioniertem Bronchoskop im Segmentbronchus.
- Interpretation beruht auf Differenzialzellbild (z. B. Lymphozytose bei Hypersensitivitätspneumonie/Sarkoidose, Eosinophilie, Neutrophilie) [6].
- Ergänzend werden Spezialfärbungen berücksichtigt, wie Hämosiderin-beladene Makrophagen bei alveolärer Hämorrhagie, PAS-positives Material bei alveolärer Proteinose oder lipidhaltige Makrophagen bei Aspiration. Besonders bei immunsupprimierten Patient:innen ist auch der gezielte Erregernachweis (insb. Pneumocystis jirovecii) von diagnostischer Relevanz.
- Selten lassen sich in der BAL auch atypische bzw. neoplastische Zellen nachweisen – vor allem im Rahmen der Abklärung unklarer Verschattungen.
- Präanalytik und zeitnahe Verarbeitung sind entscheidend.

#### EBUS-TBNA

- Echtzeit-ultraschallgestützte Nadelaspiration von mediastinalen/hilären Lymphknoten über das Bronchoskop.
- Liefert primär Zytologie plus „microcores“ für Zellblöcke.
- ≥ 3–4 Passagen und ggf. ROSE erhöhen die Ausbeute.
- Material kann als Zellblock Formalin-fixiert und in Paraffin eingebettet werden und eignet sich äquivalent Biopsien für IHC oder molekulare Analysen.

#### Transbronchiale Zangenbiopsie

- Kleine Parenchymfragmente mit teils ausgeprägten Quetschartefakten.
- Geeignet für zentrolobuläre/peribronchioläre Erkrankungen (z. B. Granulome), aber limitiert für fibrotische Muster/UIP-Architektur.
- Mehrere Proben aus verschiedenen Segmenten empfohlen.
- Im Vergleich zur Kryobiopsie meist geringere diagnostische Ausbeute und stärkere Artefakte.

#### Kryobiopsie (TBLC)

- Gefriersonde entnimmt transbronchial größere Zylinder mit erhaltener Architektur und weniger Quetschartefakten (Gefrierartefakte können durch Auftauen vor Fixierung minimiert werden).
- Bessere Beurteilbarkeit von Musterdiagnosen (z. B. UIP) und höhere diagnostische Ausbeute bei ILD in erfahrenen Zentren [7].
- TBLC wird in Leitlinien als akzeptable Alternative zur Wedge-Biopsie eingestuft (zentrumserfahren; [8]).

#### Transthorakale (CT-gestützte) Nadelbiopsie (TTNB/TTNA; Core/FNA)

- Perkutane, bildgesteuerte (meist CT) Probenentnahme vor allem von peripheren Lungenläsionen.
- Liefert zylindrische Gewebeproben (Histologie, meist architekturerhaltend) oder FNA (Zytologie/Zellblock).

### Zytologische Schnelldiagnostik vor Ort

Grundsätzlich ist es ohne weiteres möglich, direkt im Bronchoskopiesaal Ausstriche und **Tupfpräparate**

Tupfpräparate

nicht nur anzufertigen, sondern auch zu färben und zu mikroskopieren. Dies ermöglicht eine Schnellbeurteilung vor Ort („rapid on-site evaluation“, ROSE) und damit eine vorläufige Aussage über die Repräsentativität, Quantität und Qualität des gewonnen Materials. In der Indikationsstellung und Praxis existieren große Unterschiede zwischen verschiedenen Zentren. Zum Teil wird die Schnellbeurteilung von den Pneumologen selbst durchgeführt, andernorts sind es Zytotechniker oder Pathologen. Auch **telezytologische Lösungen**

telezytologische Lösungen

sind möglich. Verschiedene Studien zeigen, dass dieser Ansatz zur Verbesserung der diagnostischen Ausbeute beitragen und kosteneffizient sein kann [9].

### Digitale Diagnostik

Die digitale Diagnostik gewinnt in der Zytologie wie auch in der Histologie zunehmend an Bedeutung. In der Zytologie bieten insbesondere zeitaufwändige und zwischen Untersuchenden variabel beurteilte Analysen – wie die Differenzialzellzählung oder die **Quantifizierung**

Quantifizierung

hämosiderinhaltiger Makrophagen in der BAL – vielversprechende Ansatzpunkte für durch künstliche Intelligenz (KI) unterstützte Verfahren. Erste Studien und Publikationen verschiedener Arbeitsgruppen liegen hierzu bereits vor [10]. Wenn wir über digitale Diagnostik sprechen, ist die **PD-L1-Auswertung**

PD-L1-Auswertung

in der Histologie ein zentrales Thema. Hierfür wurden von mehreren, meist kommerziellen Anbietern Algorithmen entwickelt, die unterstützend im klinischen Alltag eingesetzt werden können.

### Standardisierung der zytologischen Diagnostik

Das neu etablierte WHO-System für Lungenzytologie wurde in einem gesonderten CME-Beitrag behandelt, auf den hier verwiesen wird [11]. Die International Collaboration on Cancer Reporting (ICCR) hat das WHO-System in ein umfassendes Protokoll zur Befundung zytologischer und kleiner histologischer Protokolle integriert, das elektronisch verfügbar ist [12]. Es kann sowohl als Checkliste dienen, als auch eine nützliche Quelle zu einer Vielzahl praktischer Fragestellungen sein.

## Interstitielle granulomatöse Lungenerkrankungen

Ein Granulom ist als Zusammenlagerung von epitheloidzelligen Makrophagen mit oder ohne Riesenzellen definiert. Bei klinischem Verdacht auf **Sarkoidose**

Sarkoidose

werden häufig neben transbronchialen Biopsien Bronchialschleimhautstufenbiopsien mit der Frage nach Granulomen eingeschickt. Falls auf den initialen Stufen keine Granulome nachweisbar sind, lohnen sich weitere Stufenschnitte, da die diagnostische Ausbeute mit rund 30 % relativ hoch ist.

In der Differenzialdiagnose zu Sarkoidose müssen bei Nachweis von Granulomen Infekte, eine **Hypersensitivitätspneumonie**

Hypersensitivitätspneumonie

oder selten auch eine Granulomatose mit Polyangiitis (GPA) bedacht werden. Hierbei spielt neben den Charakteristika der Granulome auch der Aspekt des alveolären Lungenparenchyms eine wichtige Rolle. Bei Sarkoidose liegen die konfluierenden und fibrotisierenden, **nichtnekrotisierenden Granulome**

nichtnekrotisierenden Granulome

innerhalb von nichtentzündetem Lungengewebe. Eine Hypersensitivitätspneumonie zeigt eine lymphozytäre Alveolitis und Peribronchiolitis und keine Fibrose, die Granulome sind schlechtgeformt und bestehen typischerweise aus vereinzelten interstitiell liegenden **Riesenzellen**

Riesenzellen.

Findet sich eine Fibrose um die gutgeformten Granulome, sollte eine Sarkoidose oder Infektion bedacht werden. Nekrotisierende Granulome sprechen für einen Infekt oder eine GPA. Diese zeigt typischerweise eine suppurative, „schmutzige“ Nekrose mit zahlreichen zerfallenden Neutrophilen und kann auch im Hintergrund eine organisierende Pneumonie aufweisen. Klinische Symptomatik und Radiologie können in der Abgrenzung sehr hilfreich sein.

In der BAL spricht eine **Lymphozytose**

Lymphozytose

> 20 % für eine Sarkoidose oder eine Hypersensitivitätspneumonie. In vielen Studien wird ein Schwellenwert von ≥ 25 % als aussagekräftig beschrieben, während die aktuellen Leitlinien der American Thoracic Society (ATS), der Japanese Respiratory Society (JRS) in der European Association for the Study of the Lung (ALAT) für die Diagnostik der Hypersensitivitätspneumonie einen Schwellenwert von ≥ 30 % empfehlen [6, 13, 14]. Die zusätzliche Bestimmung der **CD4-CD8-Ratio**

CD4-CD8-Ratio

wird häufig als weiteres Hilfsmittel eingesetzt: Eine erhöhte Ratio (typischerweise > 3 bis > 4) gilt für eine Sarkoidose als suggestiv, während eine erniedrigte Ratio eher mit einer Hypersensitivitätspneumonie in Verbindung gebracht wird [15, 16]. Allerdings weist dieser Parameter eine eingeschränkte Sensitivität (etwa 70 %) und Spezifität (etwa 80 %) auf, ist abhängig von Krankheitsaktivität und dem Zeitpunkt der BAL und zeigt deutliche Überschneidungen mit anderen interstitiellen Lungenerkrankungen. Daher kann er als ergänzend, aber nicht als diagnostisch beweisend betrachtet werden.

### Merke

BAL liefert essenzielle Zusatzinformationen, v. a. zur Abklärung interstitieller Lungenerkrankungen.

## Neoplastische Erkrankungen

### Atypien und Dysplasien am zytologischen Material

Obwohl in der Zytologie die Kategorie „atypische Zellen“ zur Verfügung steht, wird sie im thorakalen Bereich seltener angewendet als in anderen Organsystemen – in den meisten Fällen ist eine abschließende Diagnosestellung möglich. Die BAL stellt eine diagnostisch anspruchsvolle Situation dar, da hier atypische Zellen häufiger auftreten können. Insbesondere bei fehlendem klinischem Anhalt für eine Neoplasie und bei stark entzündlichem Hintergrund ist Vorsicht geboten, da **reaktive Atypien**

reaktive Atypien 

 – etwa von Pneumozyten – sehr ausgeprägt sein können. Ein weiterer diagnostischer Stolperstein ist die Einschätzung einer **Plattenepitheldysplasie**

Plattenepitheldysplasie

hauptsächlich im Bürstenmaterial. Hier müssen nicht nur der Grad der zytologischen Atypien und der zelluläre Hintergrund (z. B. Vorliegen von Detritus oder Nekrose), sondern auch quantitative Kriterien berücksichtigt werden. Da standardisierte Diagnosekriterien fehlen, sollte ein auf Dysplasie verdächtiger Befund immer in Zusammenschau mit den bronchoskopischen Befunden sowie gegebenenfalls mit dem parallel eingesandten histologischen Material interpretiert werden. Dabei sollte auch die Möglichkeit einer **Kontamination**

Kontamination

bzw. Verschleppung, z. B. aus den oberen Atemwegen, berücksichtigt werden. Auch hier ist eine enge Korrelation mit der Klinik erforderlich.

Eine Zusammenfassung der charakteristischen zytomorphologischen Kriterien invasiver Karzinome findet sich in Tab. 1.

**Tab. 1:** Vereinfachte Übersicht klassischer zytomorphologischer Merkmale bei Lungenkarzinomen

	Chromatin	Nukleolen	Zytoplasmagrenze	Architektur	Anderes
Adenokarzinom	Grobgranulär	Groß	Verwaschen	Gruppen (3D)	Schleim Vakuolen
Plattenepithelkarzinom	Dicht	Klein	Klar	Gruppen (2D), ev. Einzelzellen	Keratin
Kleinzelliges Lungenkarzinom (SCLC)	„Salz und Pfeffer“	Keine	–	Einzelzellen, kleine Gruppen	„Nuclear molding“

### Zytologie und kleine Biopsien – nicht alle Diagnosen erlaubt

In der aktuellen WHO-Klassifikation werden sehr hilfreiche Richtlinien an die Hand gegeben, welche Diagnosen in zytologischen Präparaten und Biopsien möglich sind und welche Diagnosen für Resektate reserviert sind, da sie entweder bestimmte prozentuale Anteile gewisser Charakteristika bedingen oder Ausschlussdiagnosen darstellen (Tab. 2). Die Diagnose NSCLC, nicht anderweitig spezifiziert (NOS), sollte zurückhaltend und nur dann eingesetzt werden, wenn keine präzise Klassifikation möglich ist. Idealerweise wird sie durch einen kurzen erläuternden Kommentar ergänzt.

**Tab. 2:** Diagnostische Terminologie bei Nachweis neoplastischer Veränderungen in Biopsien/Zytologien bzw. Resektaten der Lunge. (Modifizierte Darstellung basierend auf dem Kapitel „Small diagnostic samples“ der WHO-Klassifikation der Thoraxtumoren, 5. Auflage [17])

	Terminologie bei kleinen Biopsien/Zytologien	Terminologie bei Resektaten
Kleinzelliges Lungenkarzinom (SCLC)	Kleinzelliges neuroendokrines Karzinom	Kleinzelliges neuroendokrines Karzinom
Nichtkleinzelliges Lungenkarzinom (NSCLC)	Nichtkleinzelliges Karzinom mit neuroendokriner Morphologie und Positivität für neuroendokrine Marker, mögliches großzelliges neuroendokrines Karzinom	Großzelliges neuroendokrines Karzinom
	Sowohl squamöse als auch adenokarzinomatöse Histomorphologie: nichtkleinzelliges Karzinom, NOS (mit Kommentar)	Adenosquamöses Karzinom, wenn beide Komponenten ≥ 10 %
	Keine eindeutige Histomorphologie, aber immunhistochemisch Hinweise auf separate squamöse und adenokarzinomatöse Komponente: nichtkleinzelliges Karzinom, NOS (mit Kommentar)	Adenokarzinom, Plattenepithelkarzinom, adenosquamöses Karzinom oder großzelliges Karzinom mit unklarer Immunhistochemie
	Nichtkleinzelliges Karzinom mit Spindel- und/oder Riesenzellkomponenten (mit Kommentar)	Pleomorphes, spindelzelliges und/oder riesenzelliges Karzinom

*NOS* nicht anderweitig spezifiziert

### Immunhistochemie bei nichtkleinzelligen Lungenkarzinomen

Es ist sehr wichtig und in der WHO-Klassifikation verankert, dass im Fall von NSCLC eine nur sehr eingeschränkte immunhistochemische Diagnostik erfolgen sollte, um das Gewebe für die Analyse prädiktiver Marker zu konservieren. Bei NSCLC mit unbestimmter Morphologie bezüglich Adeno- vs. Plattenepithelkarzinom sollte die immunhistochemische Abklärung lediglich TTF‑1 und p40 umfassen [18]. Die Schwelle zum Einsatz der IHC sollte dabei jedoch grundsätzlich sehr niedrig sein, da die Morphologie täuschen kann [19]. Aufgrund des Auftretens von NSCLC mit bilineärer („bi-lineage“) Differenzierung, die trotz eindeutiger **p40-Expression**

p40-Expression

adenokarzinomtypische Driver-Mutationen zeigen können, ist eine Färbung beider Marker (TTF‑1 und p40) immer sinnvoll. Falls beide Marker negativ ausfallen, empfiehlt sich ein **Panzytokeratin**

**Abb. 3:**
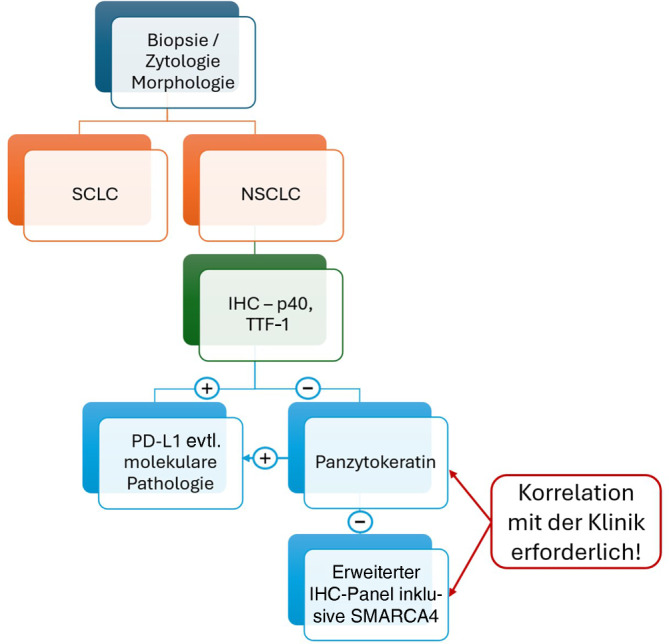
Immunhistochemie bei nichtkleinzelligen Lungenkarzinomen (*NSCLC*). *IHC* Immunhistochemie, *SCLC* kleinzelliges Lungenkarzinomen

Panzytokeratin

zur Bestätigung der Karzinomdiagnose (vs. z. B. Melanom; Abb. 3). Eine weitere immunhistochemische Abklärung sollte nur nach Rücksprache mit der Klinik im begründeten Fall einer Abklärung von **Metastasen**

Metastasen

erfolgen, z. B. GATA3 bei Mamma- oder Urothelkarzinomen. Ein Fallstrick kann hier ein Mesotheliom sein, das sich auch mit Keratinen färbt. Bei ungewöhnlicher klinischer Präsentation und ggf. ungewöhnlicher Morphologie (TTF‑1 negativ, kein Schleimnachweis) sollten auch hier weitere Färbungen durchgeführt werden. Die IHC ist in der Abklärung von Metastasen kolorektaler Karzinome vs. enterischer primärer Lungenkarzinome nicht hilfreich. Obwohl Plattenepithelkarzinome der Lunge nicht mit dem **humanen Papillomavirus**

humanen Papillomavirus

(HPV) assoziiert sind, können diese eine p16-Positivität aufweisen [20]. Eine p16-Expression sollte daher nicht als beweisend für eine Metastase eines HPV-assoziierten Plattenepithelkarzinoms des HNO- oder Genitaltrakts angesehen werden.

#### Cave

Zu breite IHC-Panels verbrauchen wertvolles Gewebe – bei NSCLC reichen TTF-1 und p40 für die Grundtypisierung.

### Kleinzelliges Lungenkarzinom

Das SCLC zeigt eine charakteristische Morphologie mit dicht gepackten, monomorphen bis leicht pleomorphen Tumorzellen mit sehr **hoher Kern-Zytoplasma-Relation**

hoher Kern-Zytoplasma-Relation,

spärlichem Zytoplasma, feingranulärem **„Salz-und-Pfeffer“-Chromatin**

„Salz-und-Pfeffer“-Chromatin,

kaum sichtbaren Nukleolen, ausgeprägtem „nuclear molding“, Quetschartefakten und meist ausgedehnter Nekrose. Azzopardi-Phänomene sind in kleinen Biopsien selten sichtbar. Eine zusätzliche Analyse der parallelen Zytologie kann von Vorteil sein, da die Morphologie hierdurch oft besser beurteilbar und eine Unterscheidung zwischen NSCLC und SCLC sowie gegebenenfalls einem Lymphom (häufig bei mediastinaler Lymphknotenbeteiligung relevant) erleichtert ist.

Aufgrund der multiplen Differenzialdiagnosen stellt das SCLC jedoch keine rein morphologische Diagnose dar [21]. Zwar ist die Morphologie typisch und kann für eine vorläufige Diagnose ausreichen, wenn die klinische Situation keinen Therapieaufschub duldet. Eine immunhistochemische Bestätigung sollte jedoch in jedem Fall erfolgen [18]. Hierzu dienen **neuroendokrine Marker**

neuroendokrine Marker

(Synaptophysin, Chromogranin A, CD56, INSM1). In etwa 80–90 % der SCLC ist TTF‑1 positiv, unabhängig vom Ausgangsorgan. Zytokeratine (v. a. das niedrigmolekulare CK8/18; CAM5.2) sind meist positiv und zeigen ein punktförmig perinukleäres („Golgi-artiges“) Muster. Selten sind SCLC zytokeratinnegativ. Fast immer liegt Ki-67 deutlich > 50 % (oft 60–80 %) und hilft v. a. bei Quetschartefakten, SCLC gegen Karzinoide abzugrenzen.

#### Cave

SCLC ist keine rein morphologische Diagnose: Immunhistochemische Bestätigung ist notwendig.

Nahezu alle SCLC weisen biallelische Inaktivierungen von *TP53* und *RB1* auf. Immunhistochemisch zeigt sich ein **Rb-Verlust**

Rb-Verlust

in etwa 90 % der Fälle [22]. In SCLC ohne eindeutige Expression neuroendokriner Marker zeigt sich häufig eine **POU2F3-Positivität**

POU2F3-Positivität

[23]. Diese Marker können bei untypischer Konstellation die Diagnose unterstützen: Ein basaloides Plattenepithelkarzinom kann CD56 oder POU2F3 positiv sein und sollte immer durch p40-Negativität ausgeschlossen sein [24]. Bei untypischem Färbemuster sollten auch ein NUT-Karzinom und ein SMARCA4-defizienter undifferenzierter Tumor immunhistochemisch ausgeschlossen werden, da sie selten ein SCLC morphologisch imitieren können.

### Prädiktive Marker inkl. Molekularpathologie

Um eine optimale Nutzung des Gewebes zu gewährleisten, wird empfohlen, schon initial von Biopsien **Leerschnitte**

Leerschnitte

zwischen den 2 gefärbten Stufen zu asservieren. Diese dienen dann der immunhistochemischen Abklärung der aktuell geforderten immunhistochemisch bestimmbaren Marker ALK, ROS1, Pan-TRK und PD-L1 und sind für eventuell zur Bestätigung durchzuführende Fluoreszenz-in-situ-Hybridisierungen (FISH) vorhanden.

Bei sehr spärlichem Gewebe kann vor der DNA-Extraktion für molekulare Analysen eine **Mikrodissektion**

Mikrodissektion

zur Anreicherung des Tumorgewebes erfolgen. Zwar wird die Analyse standardmäßig am FFPE-Material durchgeführt, jedoch stellt auch das zytologische Material aufgrund der fehlenden DNA-/RNA-Degradation durch Formalinfixierung ein exzellentes Ausgangsgewebe dar. Zunehmend wird auch, falls nötig, der zellfreie Überstand erfolgreich für die molekulare Analytik genutzt (s. zuvor; [5]).

## Fazit für die Praxis

- Kleine Biopsien und Zytologie stellen wichtige, komplementäre Materialien für die Diagnostik neoplastischer und nichtneoplastischer Lungenerkrankungen dar.
- Mit der WHO-Klassifikation liegen weltweit anzuwendende Richtlinien zur Terminologie neoplastischer Lungenerkrankungen spezifisch für die zytologische Diagnostik und Diagnose in kleinen Biopsien vor.
- Molekulare Analysen können sowohl an Biopsien als auch am zytologischen Material und am zellfreien Überstand durchgeführt werden.

## CME-Fragebogen

Welche Aussage zur Ergänzung von Zytologie und Histologie trifft am ehesten zu?

Die Zytologie ist der Histologie grundsätzlich unterlegen und daher obsolet.

Beide Methoden sind komplementär.

Die Histologie ist der Zytologie für prädiktive Marker sehr deutlich überlegen.

Die Zytologie kann kaum für die Molekularpathologie verwendet werden.

Histologie weist stets eine höhere diagnostische Ausbeute auf.

Welche Färbemethode betont typischerweise die Kerndetails und Keratinisierung auf den zytologischen Ausstrichen besonders gut?

Romanowsky

May-Grünwald-Giemsa (MGG)

Papanicolaou (PAP)

Hämatoxylin und Eosin (HE)

Ziehl-Neelsen

Wofür ist die endosonographisch gestützte transbronchiale Nadelaspiration (EBUS-TBNA) besonders geeignet?

Primäre Diagnostik peripherer Rundherde ohne Lymphknotenbefall

Gewinnung von Material im Rahmen der bronchoalveolären Lavage (BAL)

Zytologie/Zellblock aus mediastinalen/hilären Lymphknoten (Staging)

Gewinnung großer parenchymatöser Keile

Sampling aus pathologisch veränderter Bronchialschleimhaut

Bei unklarer Einordnung eines nichtkleinzelligen Lungenkarzinoms (NSCLC; Adenokarzinom vs. Plattenepithelkarzinom): Welches Immunhistochemiepanel sollte eingesetzt werden?

p40 und CK5/6

TTF‑1 und p40

TTF‑1 und Napsin A

Pan-CK und GATA3

p63 und Synaptophysin

Welche Aussage zur Immunzytochemie (ICC) trifft am besten zu?

Die ICC wird zumeist am FFPE-Material (Zellblock) durchgeführt.

Die ICC erfordert wegen variabler Vorfixierung eine eigene Validierung der Antikörper.

Die ICC ist für prädiktive Marker nur sehr eingeschränkt nutzbar.

Die ICC kann sehr häufig die Immunhistochemie (IHC) ersetzen.

ICC ist eine obsolete Methode.

Bei einem 52-jährigen Patienten mit unauffälliger Anamnese wird eine bronchoalveoläre Lavage (BAL) durchgeführt. Das BAL-Differenzialzellbild zeigt eine Lymphozytose ≥ 30 %. Woran sollte schwerpunktmäßig gedacht werden?

Exazerbation einer chronisch-obstruktiven Lungenerkrankung (COPD)

Hypersensitivitätspneumonie

Eosinophile Pneumonie

Bakterielle Pneumonie

Respiratorische Bronchiolitis

Welche Aussage zur materialschonenden Molekularpathologie ist richtig?

In der Regel werden fast ausschließlich Resektate für Next Generation Sequencing (NGS) eingesetzt.

Wegen unterschiedlicher Vorfixierungen eignen sich Zytologien oft nicht.

Der zellfreie Überstand zytologischer Proben kann verwertbare Tumor-DNA/RNA für Panelsequenzierungen enthalten.

Material aus einer endobronchialen Sonographie (EBUS) ist wegen Lymphozytenbeimischung oft ungeeignet.

Bei TTF-1-negativem nichtkleinzelligem Lungenkarzinom (NSCLC) hat eine umfassende immunhistochemische Abklärung Vorrang, eine Next Generation Sequencing(NGS)-Analyse sollte erst anschließend erwogen werden.

Welche Morphologie/Immunhistochemie (IHC) spricht am ehesten für ein kleinzelliges Lungenkarzinom (SCLC) und gegen Differenzialdiagnosen (DD)?

Große polygonale Zellen, p40+, INSM1−

Monomorphe kleine Zellen, „Salz-und-Pfeffer“-Chromatin, „nuclear molding“, INSM1/Synaptophysin+

Drüsenbildend, TTF-1−, p40+

Reichlich Zytoplasma, prominente Nukleolen, Ki-67 < 10 %

Dyskohäsiv, Panzytokeratin–, LCA+

Welche der folgenden Aussagen entspricht den WHO-Vorgaben zur Terminologie bei kleinen diagnostischen Proben?

Ein adenosquamöses Karzinom wird bereits bei Verdacht auf beide Linien diagnostiziert, ohne dass ein Mindestanteil der jeweiligen Komponenten erforderlich ist.

Die Diagnose eines großzelligen neuroendokrinen Karzinoms (LCNEC) sollte an Resektaten gestellt werden.

Enterischer Phänotyp entspricht stets einer Metastase eines kolorektalen Karzinoms

NSCLC, NOS kann großzügig verwendet werden.

Die Diagnose eines kleinzelligen neuroendokrinen Karzinoms ist an kleinen Biopsien nicht zulässig.

Bei einer 62-jährigen starken Raucherin wird eine EBUS-TBNA aus LK 10 durchgeführt, die Tumorzellen mit p40-Positivität und überwiegender TTF-1-Positivität in derselben Zellpopulation zeigt. Wie lautet Ihre Diagnose an einer Biopsie?

Plattenepithelkarzinom der Lunge

Adenokarzinom der Lunge

Nichtkleinzelliges Lungenkarzinom (NSCLC) mit „bilineärer“ („bi-lineage“) Differenzierung; gewebesparende weitere prädiktive Testung veranlassen

Metastase eines mit humanen Papillomaviren (HPV) assoziierten HNO-Tumors aufgrund von p16

Großzelliges Karzinom der Lunge
